# Managing diabetes in the psychiatric in-patient setting: knowledge, attitudes and skills of healthcare professionals

**DOI:** 10.1192/bjb.2023.70

**Published:** 2024-12

**Authors:** Zoe Goff, Vishal Sharma, Ioana Varvari

**Affiliations:** 1 Higher Trainee in Old Age Psychiatry, Leeds and York Partnership NHS Foundation Trust, Leeds, UK; 2 University of Leeds, Leeds, UK; 3 South London and Maudsley NHS Trust, London, UK

**Keywords:** Comorbidity, in-patient treatment, qualitative research, education and training, patients

## Abstract

**Aims and method:**

There is currently a lack of monitoring and standardisation of diabetes care in the National Health Service (NHS) psychiatric in-patient setting. We surveyed healthcare professionals in psychiatric in-patient units across England to understand current diabetes care. A 13-item questionnaire was piloted via think-aloud interviews. The survey was completed by healthcare professionals across 19 wards in 11 NHS mental health trusts. Results were analysed via descriptive statistics and thematic analysis.

**Results:**

Of 150 respondents, 98% agreed that addressing physical health needs was an important part of the mental health team's role; 68% agreed that they had adequate skills and knowledge to manage diabetes safely. Thematic analysis identified themes relating to individual, organisational and patient-level factors.

**Clinical implications:**

Psychiatric admission could be used opportunistically to improve the healthcare disparities for people with comorbid diabetes and severe mental illness. This national survey highlights areas that need to be addressed to optimise diabetes care in this setting.

People with severe mental illness (SMI) are at increased risk of developing diabetes, in particular type 2 diabetes.^1^ Diagnosis of diabetes has also been linked to increased risk of experiencing depression and emotional distress.^2^ It is estimated that anywhere between 2% and 25% of psychiatric in-patients have a diagnosis of diabetes.^3–5^ Suboptimal diabetes care has been associated with increased length of hospital stay^6^ and increased risk of relapse of mental illness.^7,8^

There is limited monitoring of diabetes care in the National Health Service (NHS) psychiatric in-patient setting, with the National Diabetes Audit Programme including only general practice services and physical health in-patient settings.^9^ Without clear standardisation and monitoring, managing diabetes during psychiatric admission is a missed opportunity to deliver high-quality screening and care, and ultimately help reduce disparities in accessing healthcare for this population.^10–12^

A 2017 survey of managing diabetes in people with SMI conducted among healthcare professionals across primary care and both in-patient and out-patient psychiatry found that barriers to diabetes care included lack of knowledge and training, lack of optimism about a patient's health, fear of working with people with SMI and issues with patient engagement.^13^

The aim of the national cross-sectional survey presented here was to focus specifically on the NHS psychiatric wards in England, in order to try to understand multidisciplinary healthcare professionals’ views relating to their knowledge, skills and attitudes regarding diabetes care relevant to this context, and to assess for common themes relating to the challenges these professionals face.

## Method

A 13-item questionnaire was developed to assess healthcare professionals’ knowledge, skills and attitudes regarding diabetes care in the psychiatric in-patient setting. The original framework for the questionnaire was based on National Institute for Health and Care Excellence (NICE) guidance and the training syllabuses for doctors. Additionally, separate discussions were held with two professors of psychological medicine during the development phase of the questionnaire to ensure that it met the aims of assessing knowledge, attitudes and skills before testing in interview.

The questionnaire was first piloted on a forensic rehabilitation ward via one-to-one think-aloud interviews, in order to assess its validity prior to national roll-out. Participants focused on both answering the questions and providing feedback on their interpretation and understanding of each question. The responses to the questions were not included in the final analysis, with only the interpretation assessed. Amendments were made to the questionnaire following comparison of the interview results. The final questionnaire is shown in the Supplementary material available at https://doi.org/10.1192/bjb.2023.70.

Eligibility criteria for participants included any staff member currently working in an NHS psychiatric ward in a patient-facing role. The questionnaire was circulated at local wards via site coordinators, who were recruited by email via local NHS medical education departments and psychiatry consultants with educational or academic interests.

Responses were collected primarily via anonymous electronic survey, with paper versions (also anonymous) available to boost the response rate. The aim was for a minimum of 100 responses across the 11 NHS mental health trusts; this was based on a 5% margin of error and 95% confidence interval, factoring in for drop-out and incomplete surveys. Responses were collected over a 12-week period, October to December 2021, with reminders sent in November 2021. The quantitative data were analysed via descriptive statistics and thematic analysis was conducted on qualitative data.

Qualitative data from the questionnaires were analysed using thematic analysis, based on Braun & Clarke's six-step framework.^14^ Authors Z.G. and I.V. carried out the analysis. Data were coded separately by Z.G. and I.V., for which they adopted an inductive (bottom-up) approach to identify semantic themes, using a realist approach. After the individual coding was completed, the codes were combined and discussed. As part of this, Z.G. and I.V. referred back to the data corpus to ensure that the codes were accurate and all contributions had been represented. Themes were identified using Buetow's saliency criteria, which assess the frequency (recurrence) and importance of themes.^15^ According to Buetow, themes of high importance are ‘ones that advance understanding or are useful in addressing’ the proposed question. Quotes were selected for inclusion in the results based on those that support the narrative and capture the essence of the themes. The analysis was overseen by author V.S., who is non-clinical and was able to question the validity and reliability of the analysis; however, it is understood that our personal experience may have influenced the analysis and subsequently the results presented here.

### Ethics statement

In accordance with the Health Research Authority criteria, patient records were viewed as part of NHS trust quality improvement processes, so approval from an NHS research ethics committee was not required.

### Consent statement

Responses were anonymous, confidential and did not include any patient information. Consent was therefore implied on completion of the questionnaire.

## Results

### Pilot think-aloud study

Five healthcare professionals were recruited to the pilot: two mental health nurses, one psychiatrist, one occupational therapist and one social worker, all working in the same psychiatric ward.

The primary focus of the responses was on ensuring that the wording of each question was inclusive of the range of skills and expectations for the various members of the multidisciplinary team. For example, this included amendments to the following:

- questions 8 and 9, which ask whether a professional would know what to do with a high or low blood sugar reading, to include the statement that this would be within the expectations of the professional's current role
- question 12, which asks about overall safety of diabetes care, to include information relating to both the individual's skills and knowledge and the expertise of the multidisciplinary team.

Additional amendments included ensuring that terminology was clear, such as amending ‘urine test results’ to ‘urinalysis (urine dipstick) test results’, to ensure that this was not confused with other urine tests, such as a urine drug screen.

### Descriptive statistics

A total of 156 responses were collected via the national survey (136 electronic, 20 paper). Responses came from 19 wards within 11 NHS mental health trusts across England. Geographical data were not collected as part of the survey; however, site coordinator locations included north-east, north-central, east and south-central England. Six respondents were excluded because of missing professional role information or because their roles did not involve physical healthcare. Those included in the analysis comprised 43 doctors, 55 nurses and 52 other healthcare professionals, who included dieticians, occupational therapists, pharmacists and health support workers (Table 1).

**Table 1:** Respondent characteristics

Variable	Respondents, *n* (%)
**Profession**	
Doctor	43 (28)
Nurse	55 (35)
Mental health nurse	44
Nurse consultant	1
Physical health nurse consultant	1
General nurse	1
Advanced clinical practitoner	1
Nursing associate practitioner	6
Ward manager	1
Other healthcare professional	52 (33)
Health support worker	25
Pharmacist	7
Pharmacy technician	4
Clinical psychologist	2
Assistant psychologist	1
Occupational therapist	8
Dietician	1
Social worker	1
Discharge coordinator	1
Physicians associate	1
Physiotherapy technical assistant	1
Role missing/not involving physical healthcare^a^	6 (4)
**Time in role, years**^b^	
Doctor	29
<1	4
1–2	5
2–5	7
5–10	9
10–20	2
20+	2
Nurse	46
<1	1
1–2	10
2–5	15
5–10	6
10–20	8
20+	6
Other healthcare professional	46
<1	3
1–2	7
2–5	13
5–10	5
10–20	12
20+	6
**Time since last physical health training, years**^c^	
Doctor	25
<1	7
1–2	10
2–3	3
3–4	4
4–5	0
>5	1
Nurse	26
<1	5
1–2	10
2–3	7
3–4	1
4–5	0
>5	3
Other healthcare professional	29
<1	10
1–2	11
2–3	4
3–4	2
4–5	0
>5	2

a. These six respondents were excluded from analyses.

b. Data missing for 29 respondents.

c. Data missing for 70 respondents.

**Fig. 1:**
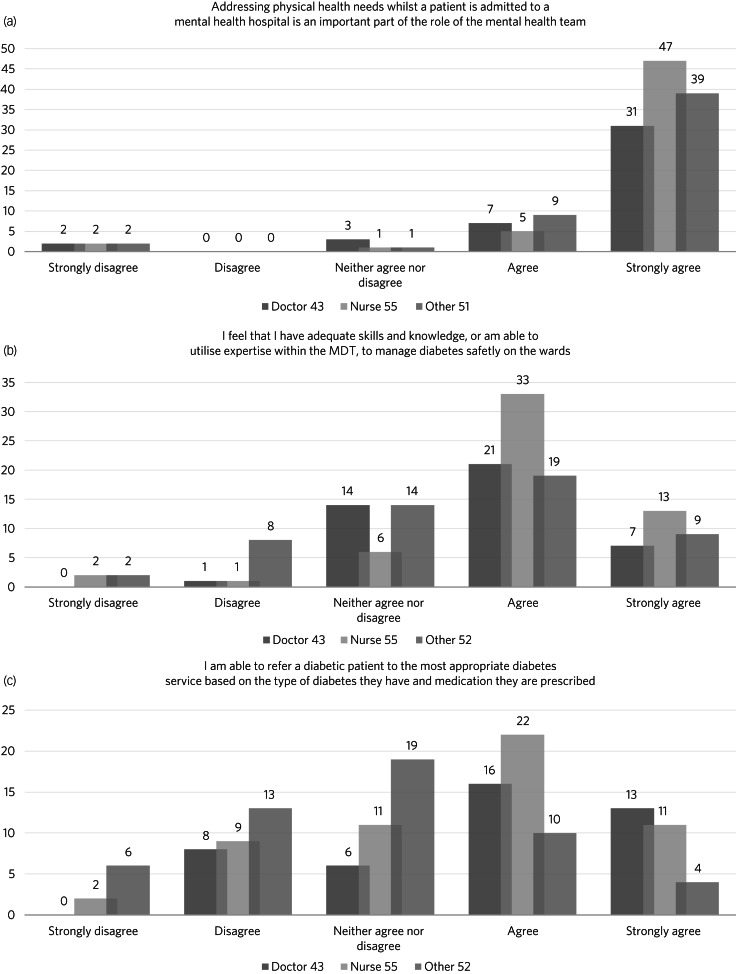
Participants’ responses to three survey questions, divided by professional group. MDT, multidisciplinary team.

In total, 93% of participants (138/148) stated that addressing physical health needs was an important part of the mental health team's role (Fig. 1(a)). However, only 28% reported having received physical healthcare training within the past 12 months. Issues relating to regular training were raised across multiple responses, including: ‘More physical health training is needed for mental health nurses’ and ‘I think there should be updates on the management of diabetes, CVD [cardiovascular disease], etc. as this moves on since psychiatrists were last in hospital’.

Overall, 68% (102/150) reported that they had adequate skills and knowledge to manage diabetes safely on the wards (Fig. 1(b)). Yet a few respondents made comments such as ‘it would be great to have direct input on the ward from a diabetes specialist nurse’, as they did not feel confident in all scenarios. In total, 51% (77/150) stated they felt able to refer a patient with diabetes to the most appropriate diabetes service based on type of diabetes and medication prescribed (Fig. 1(c)).

Overall, 69% (102/148) agreed that the diabetic care on the ward was of an acceptable standard according to NICE guidelines. However, again it was reported that advice from a specialist team about long-term treatment and medications would be valuable.

### Thematic analysis

**Fig. 2:**
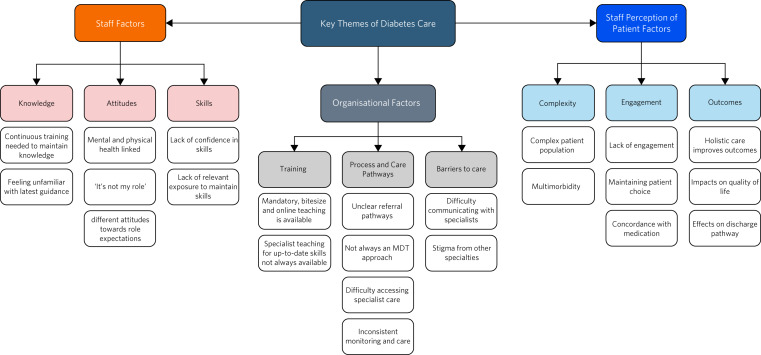
Thematic analysis map. MDT, multidisciplinary team.

The data corpus consisted of the 228 comments from staff across the 13 questions; analysis was undertaken by authors Z.G. and I.V. The analysis identified three themes and nine subthemes (Fig. 2).

#### Theme 1: organisational factors

The theme focused on three components of the organisation that have an impact on diabetes care: the training provided; processes and care pathways; and barriers to care. Respondents highlighted the lack of training pertaining to physical healthcare, such as foot care, with staff reporting, for example, that they have ‘basic knowledge’ but ‘do not feel confident’ in this area. Concerns were also highlighted in relation to existing process and care pathways: ‘I am not sure of the pathways of referral to diabetic services and from experience; the information is not always readily available’ and ‘It is not clear how/when to refer directly to diabetic clinic’.

This in turn becomes a barrier to providing adequate care: ‘It can be difficult to access diabetic nurse advice on the in-patient wards from the in-patient setting because of system limitations and lack of instructions on how to refer’ and ‘We can manage acute changes but at times we need advice from the specialist team about long term treatment and medications’. Furthermore, one person reported that delays in accessing additional support ‘puts the patient at risk’. There appeared to be a lack of ownership regarding the management of physical health needs: one respondent stated that these were ‘almost always dumped on junior doctors’, which may explain the requests for a specialist team to support the management of diabetes. Related to this, another stated ‘I can't pretend I am an expert in Diabetes, I don't want to be, I am a psychiatrist, unfortunately I end up doing everything [ … ] as no one else is interested’.

#### Theme 2: staff factors

The second theme focused more on the staff members’ knowledge, skills and attitudes regarding managing the care of a patient with diabetes. Although on the whole staff reported feeling confident in managing diabetes (‘I am probably being too hard on myself, as I would know exactly where to find the guidance for long term management, and I would be able to act in an emergency’), there was a reported lack of feeling confident (‘feeling truly confident is sometimes a state of mind that I don't feel able to reach’), and some participants reported that having access to specialist advice to support clinical decision-making would be valuable (‘it would be great to have direct input on the ward from a diabetes specialist nurse’). This was particularly the case when care related to patients with complex needs, who are undergoing long-term treatment and management of diabetes (‘at times we need advice from the specialist team about long term treatment and medications’).

#### Theme 3: staff perception of patient factors

The final theme is related to staff's perception of patient factors, specifically the complexity of the patient's needs, the level of engagement and outcomes. Generally, the participants agreed on the need to address both physical and mental health (‘physical health can have a massive impact on a person and their mental health’ and ‘patients should be assessed holistically and treated as a whole person, rather than just one aspect’), but concerns were also expressed in relation to the attention paid to physical health (‘[physical health is not always a] priority to the mental health team’). Additionally, the importance of addressing physical health needs of longer-stay patients was noted (‘[most patients’] hospital stay is for a long period and our patient cohort have physical health conditions’). An example was given by one participant about a patient who needed daily wound dressing: ‘the infection did not heal effectively [ … ]. I think the outcome would have been different if our RMNs [registered mental health nurses] could have appreciated the risks posed by diabetes for this patient’.

## Discussion

This cross-sectional survey of NHS staff highlighted that the multidisciplinary teams involved in in-patient psychiatric care generally believed that physical healthcare was an important aspect of their professional role, or at least the overall teams’ role. In particular, the qualitative component of this work demonstrated multiple attitudes relating to the importance of holistic care and the close links between good physical healthcare and mental health. Issues raised within this theme included giving equal priority to physical health concerns during a mental health admission, and how this may not always be the case, as well as considering that the length of admission to a psychiatric ward may be lengthy, so adequate physical healthcare should be provided to prevent negative effects on a person's physical health during admission. Furthermore, many raised concerns regarding the older person in-patient setting specifically, owing to issues such as complexity secondary to multiple comorbidities and the impact this can have on discharge planning.

Despite many participants focusing on the importance of physical healthcare in the psychiatric wards, there were many barriers highlighted to delivering adequate diabetes care. Although many cited utilising diabetes nurse specialist input and other community services for support, there were also concerns raised regarding clarity of referral pathways and accessing specialist diabetes advice. Issues raised included not knowing how or to whom to refer, with information on this not readily available, as well as a wish for face-to-face services in settings where only telephone advice was available. Ultimately, as provisions for accessing diabetes in-reach services and community services differ between NHS mental health trusts, it is likely that the various issues raised reflect the fact that some wards are able to readily access specialist diabetes support, whereas others are not.

Further points for discussion included issues relating to consistency of care and practical processes. These factors included inconsistent or incorrect documentation, as well as issues pertaining to consistency in monitoring and care delivered. This particularly related to capillary blood glucose monitoring, with reports of this being monitored at incorrect times or not being documented appropriately. Training was also reported to be inconsistent, which will in turn affect the knowledge and skills of professionals in this setting and is likely to result in the issues described. Lack of training was also reported to have an impact on participants’ confidence in some areas, such as diabetic foot care. In addition, there was evidence that many participants depended on skills acquired from prior experience or previous professional roles that differed from their current one, rather than any formal training. Although the ability to bring knowledge and experience is an important aspect of multidisciplinary team working, and was also in some cases cited to help in training within the team, it is also important to consider that relying on this method of knowledge sharing and training is likely to contribute to inequalities in care due to being more inconsistent than formal means of training.

Patient-related factors were also raised in many responses. These issues were multifaceted, with many raising concerns relating to the adverse influence of patients’ engagement and lifestyle factors on their physical healthcare. This included issues such as complexity secondary to multimorbidity, engagement in monitoring of, for example, blood sugars and body mass index, concordance with physical health medications, poor diet and disengagement from healthcare appointments. It is important to consider these issues as they are likely to be a key aspect of some of the barriers to delivering high-quality healthcare. Psychiatric admission could be used opportunistically to engage people with comorbid diabetes and SMI in positive healthcare behaviours, via behavioural or other appropriate interventions, which may help improve diabetes care outcomes in this population.

Overall, healthcare professionals working within the NHS psychiatric in-patient setting in England who were involved in this study consistently reported the importance of addressing physical healthcare needs, including diabetes care, during admission. Multiple barriers to high-quality and consistent care were cited, including appropriate training, clear guidelines, cohesive shared-care pathways and engagement of patients in physical healthcare monitoring and treatment. Many of the themes and barriers raised in this survey aligned with issues raised in McBain et al's 2018 survey.^13^ In particular, this included the healthcare professional's knowledge, the need for training and the difficulties of patient engagement in healthcare. These issues need to be further assessed and addressed in order to improve the diabetes care delivered within this setting.

### Limitations

Although this work provides an initial understanding of issues relating to the delivery of high-quality diabetes care in the psychiatric in-patient setting in England, further work is still needed to be able to truly understand what underlies them. This includes in-depth quantitative analysis on a national scale to be able to understand the level of implementation of NICE-endorsed care, as well as further qualitative work in the form of interviews or focus groups to provide a more detailed understanding of the barriers and enablers, at a ward level, in delivering high standards of diabetes care. Further limitations were due to the anonymity of responses: we were unable to analyse results from specific settings, such as a single ward, or to include results relating to the volume of responses per NHS trust, recurring responses within trusts or any themes that may have recurred at a ward.

## Supporting information

Goff et al. supplementary material Goff et al. supplementary material

## Data Availability

The data that support the findings of this study are available from the corresponding author, Z.G., on reasonable request.

## About the authors

**Zoe Goff** is a higher trainee in old age psychiatry working in Older Peoples Community Services with Leeds and York Partnership NHS Foundation Trust, Leeds, UK, and an Academic Clinical Fellow at the Leeds Institue of Health Sciences, University of Leeds, UK. **Vishal Sharma** is Improvement Manager and Knowledge and Evaluation Lead with Leeds and York Partnership NHS Foundation Trust, Leeds, UK. **Ioana Varvari** is a Medical Education Fellow in Psychiatry with South London and Maudsley NHS Trust, London, UK.
