# Supplementary material for: Managing diabetes in the psychiatric in-patient setting: knowledge, attitudes and skills of healthcare professionals
Source: BJPsych Bull. 2024 Dec;48(6):327–33. doi: 10.1192/bjb.2023.70 (PMC11669463; doi:10.1192/bjb.2023.70)
Supplement: Goff et al. supplementary material [file S2056469423000700sup001.docx]

Thank you for taking the time to complete this questionnaire. The questions aim to explore your knowledge, attitudes and skills relating to physical health care, particularly diabetes, in a psychiatric inpatient setting, within the context of your professional role and experience. Your response will be anonymous and confidential.

**1. What is your role on the ward? Please tick one option**

- Nurse
- Doctor
- Health support worker
- Pharmacist
- Occupational therapist
- Dietician
- Other (please specify):

**2. How long have you been in this role? Please tick one option**

- <1 year
- 1-2 years
- 2-5 years
- 5- 10 years
- 10-20 years
- 20+ years

**3. Addressing physical health needs whilst a patient is admitted to a mental health hospital is an important part of the role of the mental health team. Please tick one option**

- Strongly disagree
- Disagree
- Neither agree nor disagree
- Agree
- Strongly agree

If you would like to make any additional comments, please enter them here:

**4. Identifying and ensuring the physical health needs of patients is addressed is part of my personal job role. Please tick one option**

- Strongly disagree
- Disagree
- Neither agree nor disagree
- Agree
- Strongly agree

If you would like to make any additional comments, please enter them here:

**5. Please order these areas of physical healthcare according to how confident you feel in them, from most confident to least confident (1=most confident 6 = least confident):**

| **Areas of Healthcare** | **Rank** |
| --- | --- |
| Supporting smoking cessation |  |
| Supporting weight management |  |
| Giving dietary advice |  |
| Treatment or prevention in cardiovascular disease |  |
| Management of Type 1 or Type 2 Diabetes |  |
| Management of Alcohol and/or illicit substance use |  |

If you would like to make any additional comments, please enter them here:

**6. When did you last receive training in some aspect of the assessment or management of a physical health problem (excluding Basic Life Support and Immediate Life Support Training)? Please tick one option**

- <1 year ago
- 1-2 years ago
- 2-3 years ago
- 3-4 years ago
- 4-5 years ago
- >5 years ago

Please provide additional information about the type of training you received:

**7. I feel confident in the following physical health care responsibilities for a patient with diabetes:**

**7a) Foot care. Please tick one option.**

- Strongly disagree
- Disagree
- Neither agree nor disagree
- Agree
- Strongly agree

If you would like to make any additional comments, please enter them here:

**7b) Injection technique and injection sites. Please tick one option.**

- Strongly disagree
- Disagree
- Neither agree nor disagree
- Agree
- Strongly agree

If you would like to make any additional comments, please enter them here:

**7c) Urinalysis (urine dipstick) test results. Please tick one option.**

- Strongly disagree
- Disagree
- Neither agree nor disagree
- Agree
- Strongly agree

If you would like to make any additional comments, please enter them here:

**7d) Monitoring of blood glucose or HbA1_c._ Please tick one option_._**

- Strongly disagree
- Disagree
- Neither agree nor disagree
- Agree
- Strongly agree

If you would like to make any additional comments, please enter them here:

**8. If a patient with diabetes had a low blood sugar, within the expectations of my current role, I would know what to do. Please tick one option**

- Strongly disagree
- Disagree
- Neither agree nor disagree
- Agree
- Strongly agree

If you would like to make any additional comments, please enter them here:

**9.** **If a patient had a high blood sugar or HbA1c, within the expectations of my current role, I would know what to do. Please tick one option**

- Strongly disagree
- Disagree
- Neither agree nor disagree
- Agree
- Strongly agree

If you would like to make any additional comments, please enter them here:

**10.** **For my current role,** **I have an adequate understanding of the differences between type 1 and type 2 diabetes. Please tick one option**

- Strongly disagree
- Disagree
- Neither agree nor disagree
- Agree
- Strongly agree

If you would like to make any additional comments, please enter them here:

**11.** **I am able to refer a diabetic patient to the most appropriate diabetes service based on the type of diabetes they have and medication they are prescribed.** **Please tick one option**

- Strongly disagree
- Disagree
- Neither agree nor disagree
- Agree
- Strongly agree

If you would like to make any additional comments, please enter them here:

**12.** **Overall, I feel that I have the adequate skills and knowledge, or am able to utilise expertise within the MDT, to manage diabetes safely on the wards.** **Please tick one option**

- Strongly disagree
- Disagree
- Neither agree nor disagree
- Agree
- Strongly agree

**13. Overall, I feel that the diabetic care that patients receive on the ward I work is of an acceptable standard according to NICE Guidelines. Please tick one option**

- Strongly disagree
- Disagree
- Neither agree nor disagree
- Agree
- Strongly agree

If you would like to make any additional comments, please enter them here:
